# Miscellaneous Items

**Published:** 1857-06

**Authors:** 


### Miscellaneous Items.

☞ Vomiting in pregnancy, when not relieved by a few drops of chloroform with tincture of hyosciamus, will be found to depend in many cases upon some uterine displacement. In general the correction of the the mal-position may be easily and safely effected by the finger, and immediate relief of the emesis be obtained. The uterus may be partially retroverted, or, as in a case we have lately seen reported, be crowded into the hollow of the sacrum. [Medicel Gazette.

*Nigger Speculation.*—There is a gentleman of Louisiana (so says the Norfolk, Va. Argus) who buys niggers in the Old Dominion who are of Consumptive habits, takes them down to his sugar plantation, feeds them on tender cane shoots, which cures them and fattens them up, when he takes them to market and disposes of them at an advanced price, and then goes to work upon another stock of dilapidated darkies. This he makes a regular business of, and has a standing order in Virginia for consumptive niggers. Very likely it is a profitable speculation to him, and the niggers may be very thankful for a new hold upon life, and the continued society of Miss Dinah. But it is very like the operations of some of our "horse doctors," who buy knock-kneed and broken-winded horses, fix them up, fat them, smooth their skins, and put them into market as good as new.

☞ A friend, in speaking of California, says:—If you call a physician, he generally relieves you, if not of your disease, at least of your pocket-book. For three "ahems," and a "ha," I paid in August last, twenty-seven dollars.

